# Feasibility of C-arm guided closed intramedullary pinning for the stabilization of canine long bone fractures

**DOI:** 10.14202/vetworld.2015.1410-1415

**Published:** 2015-12-18

**Authors:** Anupreet Kaur, Ashwani Kumar, Deepesh Kumar, Jitender Mohindroo, Narinder Singh Saini

**Affiliations:** Department of Veterinary Surgery and Radiology, College of Veterinary Science, Guru Angad Dev Veterinary and Animal Sciences University, Ludhiana - 141 004, Punjab, India

**Keywords:** C-arm guided pinning, closed pinning, dog, end threaded pin, fracture

## Abstract

**Aim:**

To evaluate the feasibility of C-arm guided closed intramedullary pinning (simple Steinmann and end threaded) techniques for the stabilization of various canine long bone fractures.

**Materials and Methods:**

The present study was conducted on 19 dogs with long bone fractures which were stabilized using simple Steinmann (Group I; n=6) and end threaded (Group II; n=13) pinning under C-arm guidance. Signalment, history of trauma, clinical examination, and hematobiochemical findings were recorded at the time of presentation. Radiography of the affected limb was carried out in two views to determine type and site of the fracture. Treatment of all the fractures was attempted using simple Steinman and end threaded pinning under the C-arm guidance. The success and failure of the closed technique were correlated with age, site, and type of fractures.

**Results:**

The mean body weight and age of the dogs were 18.53±2.18 kg and 21.58±5.85 months, respectively. Early presented cases at a mean day of 2.84±0.54 were included. Out of 19 cases, it was possible to place implant successfully in 10 cases (success rate 52.63%) only. The remaining 9 cases had serious intraoperative complications like a misdirection of the pin after engaging the proximal fragment (n=3), missing the proximal fragment completely, and formation of the false tract (n=6). The majority of these complications were associated with younger age and proximal or distal third oblique fractures. High success rate of C-arm guided closed pinning was observed in midshaft fractures (75%) and transverse fractures (77.78%) in dogs of more than 1 year of age (77.78%). Simple Steinmann pinning was better feasible in a closed manner with a high success rate (66.70%) but also had implant related complications. Although, C-arm guided end threaded pinning was less (46.15%) successful, slightly tedious and time-consuming but had better implant stability than that of simple intramedullary pinning.

**Conclusions:**

From the present study, it was concluded that C-arm guided closed pinning is recommended in transverse and midshaft fractures of the long bones in dogs older than 1 year of age. Furthermore, there is need to improve traction devices for enhancing the success of C-arm guided intramedullary pinning in dogs.

## Introduction

Reduction, retention, and immobilization are the basic principles of fracture management. Reduction of long bone fractures can be achieved by closed and open methods. Reduction by the open method is associated with additional soft tissue trauma, periosteal stripping, and disturbance of hematoma at the site of fracture [1]. Hematoma formation is a necessary stage for the initiation of fracture healing [2] as it contains multilineage mesenchymal progenitor cells and has the inherent osteogenic potential [3,4].

Biological osteogenesis is a recent concept that involves adequate stabilization of fracture without interfering physiological environment at the site of fracture [5]. It can be achieved by using C-arm guided closed intramedullary pinning which is a safe and widely practiced procedure [6]. The C-arm guided closed intramedullary pinning procedure has many advantages like a minimum surgical wound, less post-operative care and less painful [6,7]. Basinger and Suber [8] opined that blood supply to the fracture was preserved, and there was minimal disruption of the fracture hematoma in closed fracture stabilization.

Intramedullary pinning is one of the most common used techniques for the repair of long bone fractures. A biomechanical study on canine femur indicated better holding the strength of partially threaded tipped over the non-threaded tipped pins [9]. In another paper, end threaded pinning has not been reported to be superior over simple Steinman pins [10]. Simple intramedullary pinning technique is associated with many implant related complication [11]. Now-a-days dynamic fracture fixation is preferred over rigid fixation [12].

It was hypothecated that C-arm guided end threaded pinning may provide sufficient stability to fracture with minimum damage to hematoma but at the same time because of the threaded portion it may be difficult to place into a medullary cavity in the closed manner. The present study was carried out to study the feasibility of C-arm guided simple Steinman and end threaded intramedullary pinning techniques for the stabilization of long bone diaphyseal fractures in dogs.

## Materials and Methods

### Ethical approval

The present study was duly approved by the Institutional Animal Ethical Committee.

### Signalment and history

The present clinical study was carried out on 19 (13 male and 6 female) dogs, presented to the Department of Veterinary Surgery and Radiology with a history of lameness following trauma and exhibiting clinical signs suggestive of the long bone fracture. The mean age and body weight of the dogs studied were 21.58±5.85 months (range 2-84 months) and 18.53±5.85 kg (range 6-40 kg), respectively. The cases were presented with a mean of 2.84±0.54 days (1-9 days).

### Clinical examination

Palpation of affected bone was done to assess crepitation and soft tissue swelling. Pre-operative radiography in mediolateral and craniocaudal views, hematology (hemoglobin, total leukocyte count and differential leukocyte count) and serum biochemistry (alkaline phosphatase, calcium, and phosphorus) was done in all the cases. General condition of the affected dog was assessed from its normal alertness, ability to walk or stand on the healthy limbs and stable cardiopulmonary parameters.

### Surgical procedure

**Figure-1:**
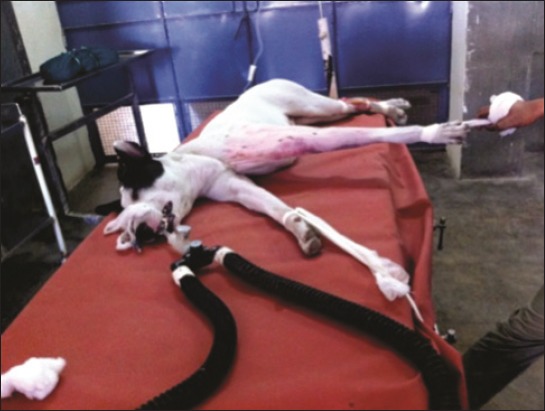
Application of traction to the forelimb with fractured humerus in the anesthetic period prior to surgery.

**Figure-2:**
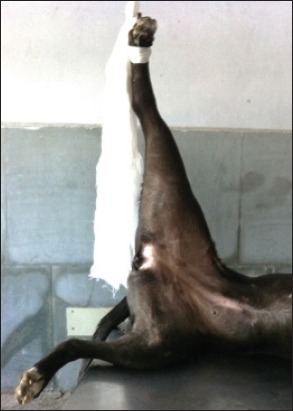
Application of traction to the hindlimb with fractured femur in the anesthetic period.

The preanesthesia for the surgical procedure comprised butorphenol (0.2 mg/kg), acepromazine (0.05 mg/kg), and glycopyrolate (0.01 mg/kg) combination intramuscularly. Anesthesia was induced with 5% thiopentone sodium, intravenously “until effect.” The following endotracheal intubation, all the patients were maintained on isoflurane (1.5-2%) with oxygen using the partial rebreathing system. For closed reduction and stabilization of fractures, a small skin incision was made for normograde pin insertion. Adequate traction was applied on the fractured limb under general anesthesia so as to reduce the fractured fragments (Figures-1 and -2).

**Figure-3:**
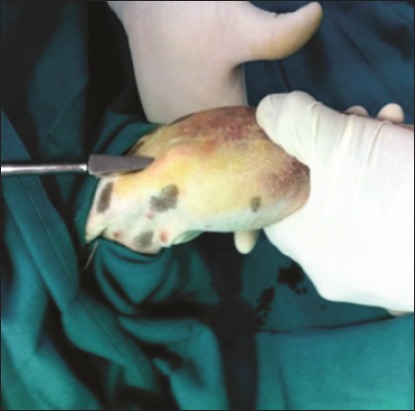
Creation of entry point of the pin for closed pinning using an awl.

**Figure-4:**
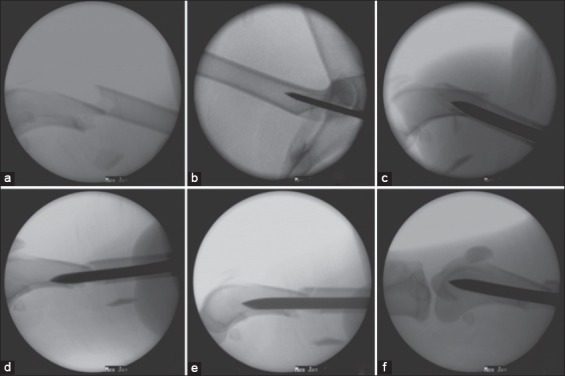
C-arm guided simple intramedullary pinning in femur. (a) Fracture site of femur, (b) Insertion of pin into the proximal end of femur, (c) Pin at the fracture site of femur, (d) Toggling of the fractured bone fragments to reduce the fracture, (e) Advancing the pin into the distal fragment of femur, (f) Seating the pin into the distal fragment.

**Figure-5:**
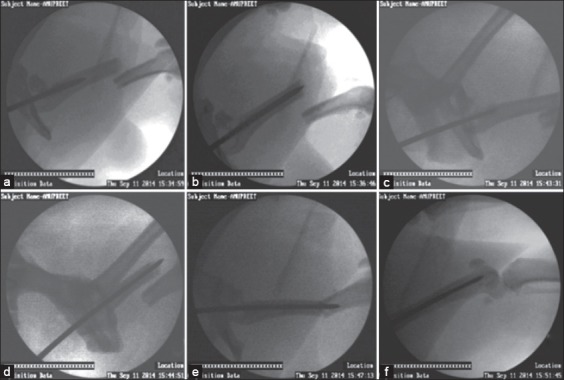
C-arm guided end threaded intramedullary pinning in femur. (a) Insertion of simple Steinmann pin into the proximal bone fragment, (b) Complete reaming of the proximal bone fragment using Simple Steinmann pin, (c) Insertion of end threaded intramedullary pin into the proximal bone fragment after withdrawing out the Simple Steinmann pin, (d) End threaded pin at the fracture site of femur, (e) Toggling of the fractured bone fragments to reduce the fracture, (f) Seating the pin into the distal bone fragment.

To stabilize humerus fracture, the pin was inserted into bone obliquely just lateral to the ridge of the greater tubercle. For femur, the greater trochanter was palpated, and the pin was inserted from the trochanteric fossa. For tibia, it was inserted at the proximal end of bone approximately ¼ inch caudomedial to the tibial tuberosity. All the fractures were attempted to stabilize using simple Steinmann (Group I; n=6) and end threaded (Group II; n=13) intramedullary pins in a closed manner. A small (1 cm) skin incision was made to locate the pin insertion site. Approximate size of the implant was selected occupying more than 50% of the diameter of the medullary canal at isthmus of the long bone which was measured using computerized radiography system, preoperatively. Using awl, the entry point for pin into the medullary canal was created (Figure-3). Then, the Simple Steinmann pin was progressed into the medullary cavity which was confirmed using C-arm visualization. The fractured fragments were toggled by manipulation. The pin was further progressed into the distal fragment and seated adequately into the distal epiphysis in Group I animals (Figure-4). In Group II, the simple Steinmann pin was withdrawn, and end threaded intramedullary pin was inserted into the medullary cavity (Figure-5). The fractures which could not be reduced and/or stabilized by closed technique were further stabilized by open method. The ratio of implant diameter and minimum diameter of the medullary cavity at the isthmus were determined from the post-operative radiographs.

### Post-operative care

Animals were given cefotaxime at 20 mg/kg twice a day and meloxicam at 0.2 mg/kg once a day for 5 and 3 days, respectively. Modified Robert-Jones bandaging was applied on the operated limb at least for 2 weeks, and owner was advised to restrict the vigorous activity of the animal. Medio-lateral and craniocaudal radiographs were taken at the immediate post-operative period and then at 2 weeks and at 4-8 weeks or whenever the case was presented for clinical and radiographic assessment or until the complete fracture healing. Removal of the implant was done after the complete fracture union was evident.

## Results and Discussions

The long bones included in the present study were femur (n=10), tibia (n=6), and humerus (n=3). The early cases of fracture with a mean ± standard error day of 3.00±0.63 in Group I and 2.76±0.75 in Group II were included. Mild or no significant swelling on the fractured limb was observed which may be attributed to simple fractures and early presentation of the cases. All sites of fractures namely proximal third (n=6), mid shaft (n=8), and distal third (n=5) were included in the present study (Table-1). The major etiology of fracture was automobile accident (36.8%) followed by fall from height (21.1%), abusive (21.1%), stuck in cot (10.5%), and unknown (10.5%). Similar findings have been observed previously [13]. In both the groups, except the fractured bone itself, none of the case had any associated skeletal injury. The distal fragment length was 6.50±2.14 cm in Group I and 7.71±0.98 cm in Group II. The mean hemoglobin level in the affected dogs were normal (9.42±0.62 g/dL) but neutrophilic (75.94±3.67%) leukocytosis (17266.84±1849.88/µL) was recorded. Leukocytosis may result from corticosteroid release due to stress, pain, anesthesia, trauma and surgical manipulation [14]. The mean value of alkaline phosphatase, calcium, and phosphorus was 162.12±13.42 IU/L, 8.97±0.47 mg/dL and 4.94±0.34 mg/dL, respectively.

**Table-1:** 

Outcome	Mid shaft (%)	Proximal third (%)	Distal third (%)
Successful	6 (75)	1 (16.67)	3 (60)
Unsuccessful	2 (25)	5 (83.33)	2 (40)
Total	8	6	5

**Figure-6:**
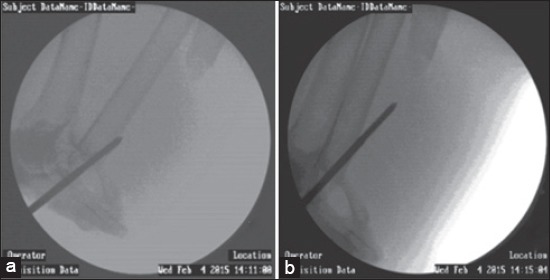
Complications of C-arm guided pinning: Missing the proximal fragment. (a) Pin missing the proximal fragment of the bone, (b) Pin missing the proximal fragment of the bone again.

**Figure-7:**
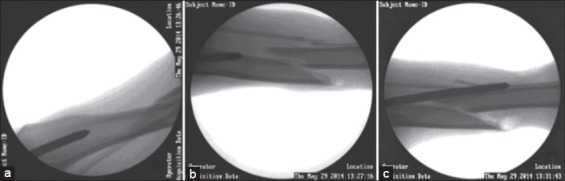
Complications of C-arm guided pinning: Misdirection of the pin and formation of false tract. (a) Pin inserted into the proximal fragment of the bone, (b) Misdirection of the pin out of the cortex of the proximal fragment, (c) Pin entering the same false tract on attempting the pinning multiple times.

In Group I, out of 6 cases closed simple pinning was successfully done in 4 cases (66.7%). In Group II, 13 cases were attempted for end threaded pinning and it could be placed successfully in 6 cases only (46.15%). While placing the end threaded in a normograde fashion, the threads of the pin gets entangled with the adjoining soft tissue structures and produces addition resistance to progression of the pin into the medullary canal. Moreover, end threaded pinning requires perfect reduction at the site of fracture before being seated properly into distal fractured fragment. But, closed pinning offer limited manipulation leading to poor reduction at the site of the fracture. These factors might be the possible reasons for reduced success of placing end threaded pin, in the present study. However, the simple Steinmann pinning was found better feasible in a closed manner compared to that of end threaded pinning. In the present study, 9 cases had intraoperative complications like a misdirection of the pin after engaging the proximal fragment (n=3) (Figure-6), missing the proximal fragment completely and formation of false tract (n=6) (Figure-7).

High success rate of closed pinning was observed in midshaft fractures (75%) compared to proximal third and distal third fractures (Table-1). Similarly, high success rate of closed intramedullary pinning was observed in transverse fractures (77.78%) while the high percentage of failure was observed in oblique fractures (Table-2). When taking the age into consideration closed pinning was more successful in dogs which were more than 1 year of age (77.78%) than dogs <1 year of age (30%) (Table-3). It was found that C-arm guided closed pinning is better feasible for skeletally mature dogs as compared to young dogs because well-developed cortex of long bones in adult animals facilitate direction of the pin within the medullary cavity. Both structural and material properties of immature bone are considerably different from those of adult bone and are characterized by lower strength, and stiffness, as well as lower yield stress and elastic modulus [15]. In the present study, using the closed method, the successful pin placement could be achieved in ≥50% fractures of each long bone (Table-4). Moreover, limited availability of effective and specific techniques and devices for fracture reduction is a major constraint of minimally invasive osteosynthesis and further improvement of the traction devices may improve the success rate of C-arm guided pinning [16].

**Table-2:** Fracture configuration wise success rate of C-arm guided intramedullary pinning.

Outcome	Transverse (%)	Oblique (%)
Successful	7 (77.78)	4 (40)
Unsuccessful	2 (22.22)	6 (60)
Total	9	10

**Table-3:** Age wise success of C-arm guided IM pinning.

Outcome	<1 year (%)	>1 year (%)
Successful	3 (30)	7 (77.78)
Unsuccessful	7 (70)	2 (22.22)
Total	10	9

**Table-4:** Bone wise success rate of C-arm guided intramedullary pinning.

Outcome	Femur (%)	Tibia (%)	Humerus (%)
Successful	5 (50)	3 (50)	2 (66.67)
Unsuccessful	5 (50)	3 (50)	1 (33.33)
Total	10	6	3

The mean±standard error intraoperative time was found to be non-significantly less in Group I (35.00±4.56 min) than in Group II (41.67±4.77 min) suggesting that C-arm guided end threaded pinning is comparatively more tedious and takes more time than simple intramedullary pinning. No wound healing related complications were seen in any of the cases. Minimal exposure of internal structures (site of fracture and adjoining soft tissue) to the external environment could be the probable reason for uneventful recovery of surgical wounds in animals of the present study. Similar observations have been reported previously [7,17]. Moreover, the risk of post-operative infection has been reported as high as twice for animals undergoing a 90 min procedure compared with those that had a 60 min procedure [18].

In the present study, out of 10 cases operated successfully by closed intramedullary pinning, three had implant related complications. In Group I, one implant was dislodged after 2 weeks and in another dog proximal migration of pin was seen [11]. Both the complications were attributed to the heavy weight of the animal; however, satisfactory fracture healing was seen in these cases. In Group II, no implant related complication was observed and fractures healed uneventfully except one in which implant was dislodged after 5 days and owner did not agree for the second surgery. Hence, comparatively a few complications were recorded in Group II which corroborates to the earlier findings that partially threaded tipped IM pins provide better holding strength than non-threaded tipped IM pins [9]. Fully threaded intramedullary pins have also been recommended for the repair of long bone fractures in dogs and cats [19,20]. Threaded pins have also been found to provide better stability in external skeletal fixators [21]. In contrast, another report did not find any advantage of end threaded pins over simple Steinman pins [10]. On long term follow-up 9 out of 10 fractures repaired by closed methods healed. In Group I, only one implant was removed and in Group II, 3 implants were removed. The remaining animals were not presented for implant removal but had satisfactory limb usage and fracture healing as recorded on telephonic follow-up.

## Conclusion

The following conclusions were drawn from the present study:

1. The C-arm guided closed pinning is recommended in transverse and mid shaft long bone fractures of the skeletally mature dogs
2. The C-arm guided closed end threaded intramedullary pinning is tedious but provides stable immobilization of fracture as compared to simple intramedullary pinning.

## Authors’ Contributions

AK, designed this clinical study under the guidance of Ashwani Kumar, JM and NSS. Ashwani Kumar, DK, AK, JM and NSS performed surgical interventions. AK and Ashwani Kumar performed post-operative care and follow-up. AK collected data, analyzed and prepared the manuscript. All the authors read, revised and approved the final manuscript.
